# Multiple Fungal Metabolites Including Mycotoxins in Naturally Infected and *Fusarium*-Inoculated Wheat Samples

**DOI:** 10.3390/microorganisms8040578

**Published:** 2020-04-17

**Authors:** Valentina Spanic, Zorana Katanic, Michael Sulyok, Rudolf Krska, Katalin Puskas, Gyula Vida, Georg Drezner, Bojan Šarkanj

**Affiliations:** 1 Agricultural Institute Osijek, Juzno predgradje 17, 31000 Osijek, Croatia; georg.drezner@poljinos.hr; 2 Department of Biology, Josip Juraj Strossmayer University of Osijek, Cara Hadrijana 8a, 31000 Osijek, Croatia; zorana.katanic@biologija.unios.hr; 3 Institute of Bioanalytics and Agro-Metabolomics, Department of Agrobiotechnology (IFA-Tulln), University of Natural Resources and Life Sciences Vienna (BOKU), Konrad Lorenzstr. 20, 3430 Tulln, Austria; michael.sulyok@boku.ac.at (M.S.); rudolf.krska@boku.ac.at (R.K.); 4 Institute for Global Food Security, School of Biological Sciences, Queen’s University Belfast, University Road, Belfast BT7 1NN, Northern Ireland, UK; 5 Agricultural Institute, Centre for Agricultural Research, Brunszvik u. 2, 2462 Martonvásár, Hungary; puskas.katalin@agrar.mta.hu (K.P.); vida.gyula@agrar.mta.hu (G.V.); 6 Department of Food Technology, University Centre Koprivnica, University North, Trg dr. Žarka Dolinara 1, 48000 Koprivnica, Croatia; bsarkanj@unin.hr

**Keywords:** fusarium, LC-MS/MS, mycotoxin, occurrence, wheat

## Abstract

In this study, the occurrence of multiple fungal metabolites including mycotoxins was determined in four different winter wheat varieties in a field experiment in Croatia. One group was naturally infected, while the second group was inoculated with a *Fusarium graminearum* and *F. culmorum* mixture to simulate a worst-case infection scenario. Data on the multiple fungal metabolites including mycotoxins were acquired with liquid chromatography with mass spectrometry (LC-MS/MS) multi-(myco)toxin method. In total, 36 different fungal metabolites were quantified in this study: the *Fusarium* mycotoxins deoxynivalenol (DON), DON-3-glucoside (D3G), 3-acetyldeoxynivalenol (3-ADON), culmorin (CULM), 15-hydroxyculmorin, 5-hydroxyculmorin, aurofusarin, rubrofusarin, enniatin (Enn) A, Enn A1, Enn B, Enn B1, Enn B2, Enn B3, fumonisin B1, fumonisin B2, chrysogin, zearalenone (ZEN), moniliformin (MON), nivalenol (NIV), siccanol, equisetin, beauvericin (BEA), and antibiotic Y; the *Alternaria* mycotoxins alternariol, alternariolmethylether, altersetin, infectopyron, tentoxin, tenuazonic acid; the *Aspergillus* mycotoxin kojic acid; unspecific metabolites butenolid, brevianamid F, cyclo(L-Pro-L-Tyr), cyclo(L-Pro-L-Val), and tryptophol. The most abundant mycotoxins in the inoculated and naturally contaminated samples, respectively, were found to occur at the following average concentrations: DON (19,122/1504 µg/kg), CULM (6109/1010 µg/kg), 15-hydroxyculmorin (56,022/1301 µg/kg), 5-hydroxyculmorin (21,219/863 µg/kg), aurofusarin (43,496/1266 µg/kg). Compared to naturally-infected samples, *Fusarium* inoculations at the flowering stage increased the concentrations of all *Fusarium* mycotoxins, except enniatins and siccanol in Ficko, the *Aspergillus* metabolite kojic acid, the *Alternaria* mycotoxin altersetin, and unspecific metabolites brevianamid F, butenolid, cyclo(L-Pro-L-Tyr), and cyclo(L-Pro-L-Val). In contrast to these findings, because of possible antagonistic actions, *Fusarium* inoculation decreased the concentrations of the *Alternaria* toxins alternariol, alternariolmethylether, infectopyron, tentoxin, tenuazonic acid, as well as the concentration of the nonspecific metabolite tryptophol.

## 1. Introduction

Wheat is the basic staple food and its global consumption is about 66 kg/per capita worldwide [1]. Various diseases can affect the heads of wheat, and severe infection can result in decreased grain yield and quality. Furthermore, wheat and its products can be contaminated with mycotoxins produced by different fungi that can be found in the field and/or postharvest [2]. Among the most important risks associated with cereal consumption are mycotoxins, heavy metals, pesticide residues, and alkaloids. It is very important to monitor mycotoxins in all stages of wheat production from the field to end-use quality usage. Fusarium head blight (FHB), caused by several *Fusarium* species, mainly *Fusarium graminearum*, *F. culmorum*, and *F. avenaceum*, is a devastating disease of wheat, associated with mycotoxin contamination with a significant threat to animal and human health. The most important mycotoxins in wheat are mainly *Fusarium* toxins, such as deoxynivalenol (DON), with its acetylated forms (15 acetyl-deoxynivalenol or 15AcDON, and 3 acetyl-deoxynivalenol, or 3AcDON), zearalenone (ZEN), nivalenol (NIV), fumonisins (FB), T-2, and HT-2 toxins and its modified forms [2,3,4]. Beside *Fusarium* spp., species from the genera *Alternaria*, *Penicillium*, and *Aspergillus* are critical in the maintenance of food safety and can cause mycotoxin contaminations of cereals [5,6]. For example, *Aspergillus flavus* can infect wheat grain in the field but also contaminates stored grains when temperature and water activity are favorable [7].

The most common mycotoxin in wheat and wheat-based products in the European Union (EU) is the well-known type-B trichothecene, DON [8]. Moreover, a very limited number of mycotoxins are subject to legislation and regular monitoring. As far as cereals are concerned, aflatoxins, fumonisins, DON, zearalenone (ZEN), and ochratoxin A (OTA) are those most often analyzed [9]. For these reasons, the other mycotoxins, which until now have not received detailed scientific attention, are commonly indicated as ‘novel’ or ‘emerging’ mycotoxins [10,11]. Therefore, there is an urgent need to acquire data on the presence and diffusion of these emerging mycotoxins in field crops, in relationship to different climatic conditions, in order to perform proper risk characterization, risk assessment, and afterwards propose maximum limits in the food chain. The European Union has established maximum levels for DON [12], where unprocessed wheat, cereal flour, bread and wheat-based foods for infants and young children must not contain more than 1250, 750, 500, and 200 μg/kg of DON, respectively. DON is known to cause food refusal, vomiting, and depressed immune function, resulting in poor weight gain [13]. Culmorins (CULMs) are tricyclic sesquiterpene diols that can be produced by *F. culmorum*, *F. graminearum*, and *F. venenatum* [14]. Nevertheless, CULM is considered an “emerging mycotoxin” whose synthesis and toxicology will be of greater interest for food safety consideration in the future [11]. It was also confirmed that CULM suppresses one of the important steps of DON detoxification—glucuronidation [15] and therefore can increase DON’s toxic effect if it co-occurs with DON. Genes for CULM and trichothecene production co-occur in other fungal species closely related to *F. graminearum* [16]. Nivalenol (NIV) to some extent co-occurs with DON. Although NIV is less toxic to plants compared to DON, it has more severe toxic effects in animals and humans [17]. ZEN is often co-produced with DON by *Fusarium* spp. such as *F. graminearum* [18]. Moreover, recent studies revealed an increased presence of modified *Fusarium* mycotoxins and so-called emerging mycotoxins, particularly enniatins (Enns), beauvericin (BEA), and moniliformin (MON) [19,20] which are far less investigated. Enns can be produced by several fungal species including *Fusarium* spp., and the enniatin analogs enniatin A (EnnA), enniatin A1 (EnnA1), enniatin B (EnnB), enniatin B1 (EnnB1), and enniatin B2 (EnnB2) are reported to be the most prevalent ones in cereals in Europe [21,22]. Beauvericin (BEA) can co-occur with Enns, since they can be produced by the same *Fusarium* species [21]. Moniliformin (MON) often was found in *Fusarium*-damaged durum wheat grains due to *F. avenaceum* infection [23,24]. The European Union maximum limits for other *Fusarium* toxins (ZEN and fumonisins B1 and B2, and T-2 and HT-2 toxins) in cereals and cereal-based products have been established by Commission Regulation [12]. Different reference points were established for mycotoxins as follows: the benchmark dose lower confidence limit of 10% (BMDL10) extra risk for aflatoxin B1 at 170 ng/kg per body weight (bw) per day; tolerable daily intake (TDI) of 2 µg/kg bw per day for fumonisins B1, B2, and B3; tolerable weekly intake (TWI) of 0.1 µg/kg bw per week for OTA; BMDL05 of 200 µg/kg bw per day for MON; and 90 µg/kg bw per day of BEA was established as a concentration with a low risk (for genotoxic carcinogens such as aflatoxins) or no risk (for other mycotoxins) [25]. The control of *Fusarium* fungal infection in the field by growing resistant cultivars could be the most efficient method to control FHB [16] and mitigating mycotoxin accumulation in the end-use products. To reduce the risk of *Fusarium* contamination, the application of preventive agricultural practices is also important, such as crop selection, rotation, tillage, irrigation, and the proper use of fungicides, as partial control of FHB [26,27]. Furthermore, mycotoxin production by mycotoxigenic fungal species is dependent on water activity, temperature, and CO_2_ levels [28,29].

In addition to these better-known compounds, other secondary metabolites produced by *Fusarium* species may be detected and investigated using multi-analytic methods developed in recent years based on liquid chromatography coupled to mass spectrometry [30,31]. Modified and emerging mycotoxins which cover DON derivatives (DON-3-glucoside, acetyl-DONs, nor-DONs and deepoxy-DON), nivalenol, T-2 and HT-2 toxins, enns, BEA, moniliformin, and fumonisins are not regulated by EU law. Furthermore, multiple fungal metabolites including mycotoxins are frequently observed [32,33]. This is a topic of great concern, as co-contaminated samples might still exert adverse health effects due to additive/synergistic interactions of the mycotoxins.

This research summarizes the occurrence of the mycotoxins/metabolites produced by *Fusarium*, *Alternaria,* and *Aspergillus*, as well as several unspecific metabolites in naturally-infected and *Fusarium*-inoculated wheat samples. To our best knowledge, the majority of mycotoxins/metabolites analyzed in this study are poorly characterized [6], and their occurrence and concentration in commercially produced wheat varieties are mainly unexplored.

## 2. Materials and Methods

### 2.1. Plant Material and Field Trial

The entire field experiment was conducted from October 2018 to July 2019 at Osijek (45°32′ N, 18°44′ E), Croatia. The soil type is a eutric cambisol. The average annual precipitation in the growing season is 531.3 mm, and the average annual temperature is 10.9 °C. Plots consisted of eight row plots with a 2.5 m length and a 1.08 m width at a sowing rate of 330 seeds/m^2^. The weed control was conducted with the herbicide Sekator (100 g/L amidosulfuron and 25 g/L iodosulfuronmethyl-sodium) at wheat tillering (GS 31). A total of 170 kg N/ha was applied to the plots as a granular ammonium nitrate fertilizer and was split between GS 23 and 45. The experiment was conducted with two experimental replications in two treatments (trials): naturally-infected and *Fusarium*-inoculated. In each treatment, the same four winter varieties were used (Ficko and Pepeljuga originated from the Agricultural Institute Osijek, Croatia; Mv Karizma and Mv Kolompos originated from the Agricultural Institute in Martonvasar, Hungary). The grains were taken by harvesting the whole plot with a Wintersteiger cereal plot combine-harvester. The harvested grains were mixed thoroughly from two replications in each treatment, and 100 g grain samples were taken from each plot to analyze the mycotoxin content.

### 2.2. Inoculum Production and Inoculation Procedure

The *Fusarium* species used in this experiment were two the most prevalent casual agents of FHB: *F. graminearum* strain (PIO 31), previously isolated from winter wheat collected in East Croatia and *F. culmorum* strain (IFA 104), obtained from IFA, Tulln. Conidial inoculum of the *F. graminearum* was produced in mung bean medium [34], while *F. culmorum* spores were produced by a mixture of wheat and oat grains [35]. Conidial concentrations of both species were determined using a hemocytometer and were set to 1 × 10^5^ mL^−1^. The spore suspensions were set to a concentration so that a single bottle of one strain contained a sufficient amount of suspension (>900 mL), which could be diluted in 100 L of water immediately before inoculation (100 mL/m^2^). One treatment was grown according to standard agronomical practice with no usage of fungicide and without misting treatment, while another treatment was subjected to two inoculation events using a tractor-back sprayer with *Fusarium* spp. at the time of flowering (Zadok’s scale 65) [36]. Misting was provided by spraying with a tractor back-sprayer on several occasions.

### 2.3. Mycotoxin LC-MS/MS Analyses

Determination of mycotoxins with the LC-MS/MS method in wheat grains and wheat malt was performed as previously described [31]: In brief, 5.00 g of ground wheat was extracted with 20 mL of extraction solvent composed of acetonitrile (AcN):water (W):acetic acid (HAC) = 79:20:1 (*v*:*v*:*v*) on a rotary shaker (GFL 3017, GFL; Burgwedel, Germany) for 90 min at room temperature in a horizontal position. After extraction, 500 µL of the extract was diluted with 500 µL of dilution solvent composed of AcN:W:HAC = 20:79:1 in vials. Finally, 5 µL was injected into an LC-MS/MS system composed of a QTrap 5500 MS/MS (Sciex, Foster City, CA, USA) coupled with an Agilent 1290 series UHPLC system (Agilent Technologies, Waldbronn, Germany). The separation of analytes was performed on a Gemini C18 column (150 × 4.6 mm i.d., 5 µm particle size) with a 4 × 3 mm precolumn with the same characteristics (Phenomenex, Torrance, CA, USA). The eluents used were composed of methanol (MeOH):W:HAC = 10:89:1 (*v*:*v*:*v*) as eluent A, and MeOH:W:HAC = 97:2:1 (*v*:*v*:*v*) as eluent B. The analysis was performed on the fully validated method described in detail by Sulyok et al. (2020) for measurement of 500+ mycotoxins and other secondary metabolites.

### 2.4. Statistical Analysis

The data were evaluated for the distribution by the Shapiro–Wilk W-test, and the homoscedasticity was determined by Levene’s test. Since the data did not show a normal distribution, the exact comparison between the two groups (naturally-infected and inoculated) was tested by the Mann–Whitney U test. The comparison of the differences in the data distribution between different tested varieties was performed by Kruskal–Wallis ANOVA. Statistical tests and boxplot graphs were performed in Statistica 13.1. (TIBCO Software Inc., Palo Alto, CA, USA), shown in the supplementary file. Graphs were prepared by using the statistical software GraphPad Prism 5.0 (GraphPad Software Inc., San Diego, CA, USA).

## 3. Results

### 3.1. Fusarium Metabolites/Mycotoxins in Fusarium-Inoculated and Naturally-Infected Samples

**Figure 1:**
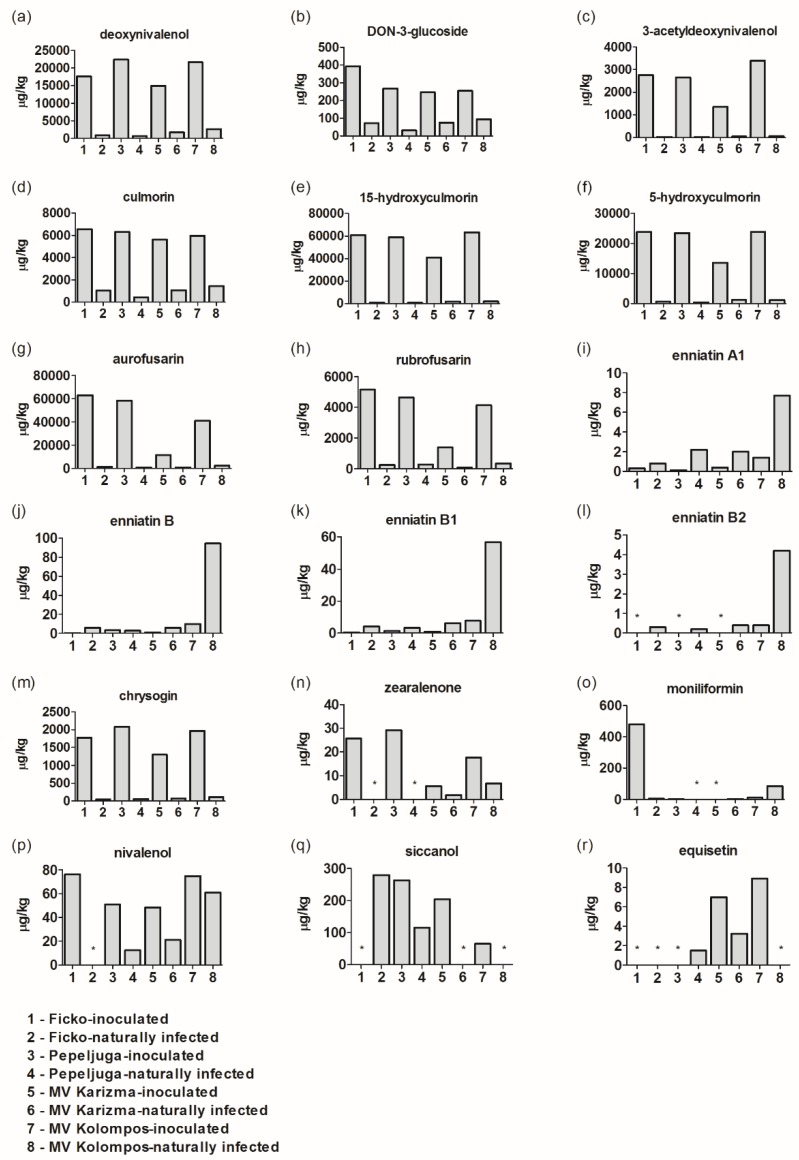
*Fusarium* mycotoxins/metabolites in *Fusarium*-inoculated and naturally-infected samples: deoxynivalenol (**a**), DON-3-glucoside (**b**), 3-acetyldeoxynivalenol (**c**), culmorin (**d**), 15-hydroxyculmorin (**e**), 5-hydroxyculmorin (**f**), aurofusarin (**g**), rubrofusarin (**h**), enniatin A1 (**i**), enniatin B (**j**), enniatin B1 (**k**), enniatin B2 (**l**), chrysogin (**m**), zearalenone (**n**), moniliformin (**o**), nivalenol (**p**), siccanol (**q**), and equisetin (**r**). The asterisk (*) indicates < LOD.

Deoxynivalenol (DON), DON-3-glucoside (D3G), 3-acetyldeoxynivalenol (3-ADON), culmorin (CULM), 15-hydroxyculmorin, 5-hydroxyculmorin, aurofusarin, rubrofusarin, enniatin (Enn) A1, B, B1 (Figure 1a–k) were found in all tested samples, although sometimes in ppb concentrations, while enniatin (Enn) B2 was present in at least half of the tested samples (Figure 1) Chrysogin was also found in all tested samples (Figure 1m), while zearalenone (ZEN), moniliformin (MON), nivalenol (NIV), siccanol, and equisetin were present in some of the tested samples (Figure 1n–r).

**Figure 2:**
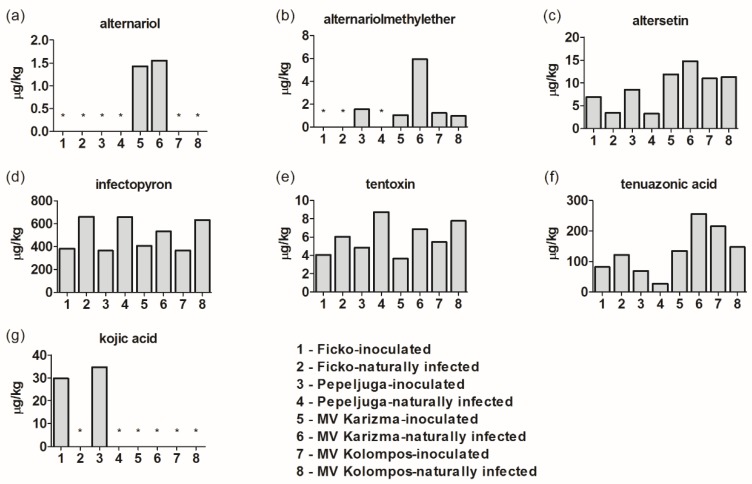
*Alternaria* and *Aspergillus* mycotoxins in *Fusarium*-inoculated and naturally-infected samples: alternariol (**a**), alternariolmethylether (**b**), altersetin (**c**), infectopyron (**d**), tentoxin (**e**), tenuazonic acid (**f**), and kojic acid (**g**). The asterisk (*) indicates < LOD.

The concentrations of *Fusarium* mycotoxins DON, CULM, 3-ADON, 15-hydroxyculmorin, D3G, and 5-hydroxyculmorin were significantly elevated in the inoculated samples compared to naturally-infected samples of all tested winter wheat varieties (Supplementary Figure S1, Figure 1a–f). For other detected *Fusarium* metabolites, i.e., aurofusarin, rubrofusarin, and chrysogin, significant increases also occurred in inoculated samples compared to those that were naturally infected (Supplementary Figure S2, Figure 2c,d,f). The levels of DON measured in four FHB-inoculated samples were 17,586 µg/kg in Ficko, 22,337 µg/kg in Pepeljuga, 14,946 µg/kg in Mv Karizma, and 21,622 µg/kg in Mv Kolompos. Although FHB-inoculated samples were far more contaminated with DON, naturally-infected samples also contained higher levels of DON, as well as several other *Fusarium* mycotoxins, than expected (Figure 1a). Moreover, in Mv Karizma and Mv Kolompos, recorded DON concentrations were 1764 µg/kg and 2682 µg/kg, respectively, which is above the maximal allowed concentration for human consumption. As expected, D3G and 3-ADON were found in all samples that contained DON (Figure 1b,c). The highest concentration of D3G was recorded in Ficko in the inoculated samples (393 µg/kg), while the lowest concentration was in naturally-infected samples of Pepeljuga (31 µg/kg). 3-ADON concentration ranged from 9 µg/kg in naturally-infected samples to 3399 µg/kg in *Fusarium*-inoculated samples. FHB-inoculated samples of Mv Karizma had at least 2 times lower concentrations of 3-ADON compared to inoculated samples of other varieties.

The highest concentration of CULM (6551 µg/kg) was recorded in FHB-inoculated samples of Ficko, and the lowest was obtained in naturally-infected samples of Pepeljuga (443 µg/kg) (Figure 1d). The highest concentrations of 15-hydroxyculmorin and 5-hydroxyculmorin were found in FHB-inoculated samples of Mv Kolompos—63,329 and 23,864 µg/kg, respectively—similar to the levels detected in inoculated samples of Ficko and Pepeljuga, but lower levels were detected in inoculated samples of Mv Karizma (Figure 1e,f).

In this study, pigments were also detected; aurofusarin was detected in the range of 735 to 63,098 µg/kg and rubrofusarin from 65 to 5136 µg/kg. Although aurofusarin and rubrofusarin levels in all inoculated samples increased compared to naturally-infected samples, inoculated samples of Mv Karizma had at least three times lower concentrations of these mycotoxins compared to other varieties (Figure 1g,h).

The highest level of enniatins A1, B, B1, and B2 (8, 94, 57, and 4 µg/kg, respectively) were found in naturally-infected samples of Mv Kolompos. Overall, these mycotoxins were shown to be present in lower concentrations in FHB-inoculated samples compared to naturally-infected samples or were not detected at all in the inoculated samples (Figure 1i–l). The various enniatins were ranked as follows, in descending order of incidence and mean concentration: enniatin B > enniatin B1 > enniatin A1 > enniatin B2 > enniatin A > enniatin B3. Total enniatin levels varied from traces (0.02 μg/kg, enniantin A) to 94 μg/kg (enniantin B).

Chrysogin, in the inoculated samples compared to those that were naturally infected, decreased by 97, 97, 94, and 94%, respectively, in Ficko, Pepeljuga, Mv Karizma, and Mv Kolompos (Figure 1m).

All inoculated and two naturally-infected samples were contaminated with ZEN, and the highest concentration of this mycotoxin was detected in FHB-inoculated samples (26 µg/kg). No ZEN was detected in naturally-infected samples of Ficko and Pepeljuga, and all detected concentrations were below legal limits set in the EU by the European Commission regulation 1881/2006 (Figure 1n).

In the case of MON, the concentration in the inoculated sample of Ficko was 480 µg/kg, which is a 99% increase in relation to naturally-infected grain, while other varieties were far less contaminated with MON, and its concentration in other tested samples ranged from 3 (Pepeljuga, inoculated sample) to 86 µg/kg (Mv Kolompos, inoculated sample) (Figure 1o).

Nivalenol (NIV) was found in seven out of eight samples. Here, the inoculated samples had increased NIV levels compared to the naturally-infected grains, although the difference between naturally-infected and inoculated samples was the least pronounced for Mv Kolompos, in which high values of NIV were detected in both treatments (Figure 1p). The differences between treatments were not statistically significantly different (data not shown).

Inoculated sample of Ficko and naturally-infected samples of Mv Karizma and Mv Kolompos had no siccanol. The highest amount was recorded in naturally-infected samples of Ficko (300 µg/kg) (Figure 1q). The presence of siccanol is not specific for *Fusarium* strains used in this research, and such a high concentration in naturally-infected sample likely came from natural co-contamination by other *Fusarium* species.

Equisetin was detected only in samples of Mv Karizma, both FHB-inoculated and naturally-infected, as well as in naturally-infected samples of Pepeljuga and inoculated samples of Mv Kolompos (Figure 1r).

In addition to the mycotoxins presented in Figure 1, beauvericin, enniatin A, fumonisin B1, fumonisin B2, and antibiotic Y were also detected in the same samples, while enniatin B3, which was tested, was not detected in any of the analyzed samples. Concentrations of beauvericin and enniatin A were very low: 0.5 μg/kg of beauvericin was detected in inoculated samples of Ficko and enniatin A was detected in naturally-infected samples of Pepeljuga, Mv Karizma, and Mv Kolompos at concentrations of 0.5 μg/kg, 0.1 μg/kg, and 0.2 μg/kg, respectively. The inoculated sample of Pepeljuga was the only variety in which fumonisin B1 and B2 were detected at concentrations of 54 μg/kg and 15 μg/kg, respectively, while antibiotic Y was only detected in inoculated samples of Mv Karizma at a concentration of 73 μg/kg. The presence of those metabolites was not specific for *Fusarium* strains used in this research and probably resulted from natural co-contamination by other *Fusarium* species. T2 and HT2 toxins were absent in those tested wheat varieties in both treatments.

### 3.2. Alternaria and Aspergillus Metabolites/Mycotoxins in Fusarium-Inoculated and Naturally-Infected Samples

Concentrations of six *Alternaria* and *Aspergillus* metabolites/mycotoxins in *Fusarium*-inoculated and naturally-infected samples of four wheat varieties are presented in Figure 2. Alternariol was detected only in variety Mv Karizma, in both inoculated and naturally-infected samples (Figure 2a). The highest concentration of alternariolmethylether was detected in naturally-infected Mv Karizma (6 µg/kg), while naturally-infected Pepeljuga and Ficko in both treatments had no alternariolmethylether (Figure 2b). Altersetin, infectopyron, tentoxin, and tenuazonic acid were detected in all varieties in both treatments (Figure 2c–f). In naturally-infected samples, compared to inoculated samples, the mean infectopyron and tentoxin concentrations were significantly increased (Supplementary Figure S2, Figure 2a,b). The *Aspergillus* metabolite kojic acid was found only in FHB-inoculated samples of Ficko and Pepeljuga (Figure 2g).

### 3.3. Other Metabolites in Fusarium-Inoculated and Naturally-Infected Samples

**Figure 3:**
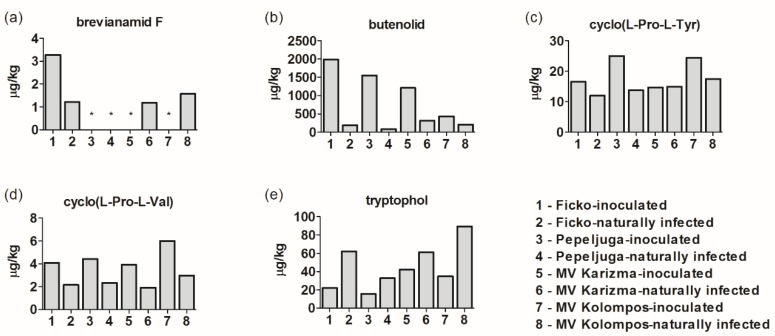
Other metabolites in *Fusarium*-inoculated and naturally-infected samples: brevianamid F (**a**), butenolid (**b**), cyclo(L-Pro-L-Tyr) (**c**), cyclo(L-Pro-L-Val) (**d**), and tryptophol (**e**). The asterisk (*) indicates < LOD.

Concentrations of additional five unspecific metabolites in *Fusarium*-inoculated and naturally-infected samples are presented in Figure 3. Brevianamid F was found in both treatments in Ficko, and in naturally-infected samples of Mv Karizma and Mv Kolompos (Figure 3a). Butenolid was found in all samples with increased concentrations in inoculated samples compared to naturally-infected samples (Figure 3b) (Supplementary Figure S2, Figure 2e). Cyclo(L-Pro-L-Tyr) and cyclo(L-Pro-L-Val) were increased in inoculated samples (Figure 3c,d), while tryptophol had higher concentrations in naturally-infected samples (Figure 3e).

There were no observed statistical differences between different wheat varieties tested by Kruskal–Wallis ANOVA (data not shown).

## 4. Discussion

This study presents the first report on the occurrence of 36 fungal metabolites in the wheat grain of four different wheat varieties from Croatia and Hungary and their concentrations after artificial inoculation with *Fusarium* spp. compared to non-inoculated field-grown wheat plants.

The major mycotoxins occurring in wheat, at levels of potential concern for human and animal health, are *Fusarium* mycotoxins [2]. *F. graminearum* occurs worldwide as well as in Croatia [37] and is the most important producer of deoxynivalenol (DON), type B trichothecene. The most frequently detected mycotoxins in wheat grain are DON, FB, ZEN, produced by *Fusarium* species, and aflatoxins (AFs) and ochratoxin A (OTA) produced by *Aspergillus* and *Penicillium* species, respectively [38]. In temperate areas, DON is the most prevalent mycotoxin in wheat [39]. Field survey reports clearly indicate that the mycotoxins most frequently produced in cereal head blight by *F. graminearum* and *F. culmorum* in all European countries are DON, 3-acetyldeoxynivalenol (3-ADON), and zearalenone (ZEN) [40].

Several studies reported a high incidence of multi-mycotoxin contamination in cereals and agricultural commodities [41]. The current investigation showed that in 2019, wheat samples in eastern Croatia were co-contaminated by 24 *Fusarium* mycotoxins/metabolites. A study carried out in Italy showed that at least 80% of wheat samples were contaminated with one mycotoxin, while two mycotoxins were found in 27% of contaminated samples; 38% of the analyzed samples were contaminated with three or more mycotoxins [42].

### 4.1. Deoxynivalenol (DON), DON-3-Glucoside (D3G), and 3-Acetyldeoxynivalenol (3-ADON)

As regards the contamination levels, the obtained results showed that mycotoxins such as DON, D3G, and CULM, which are mainly produced by *F. culmorum* and *F. graminearum*, were the most abundant mycotoxins in the environmental conditions of the considered field experiments. Average levels of DON contamination did not exceed risk threshold levels for Ficko and Pepeljuga in naturally-infected samples, but as the content range was very wide, all *Fusarium*-inoculated and two naturally-infected (Mv Karizma and Mv Kolompos) samples exceeded the maximum levels for DON contamination. The mean and maximum DON concentrations of naturally-infected wheat samples are summarized in Figure 1 and are lower than the maximum DON concentrations reported in the European wheat summary—a concentration as high as 10,000 µg/kg DON was reported in the European results [8].

Wheat samples had a high incidence of 3-ADON, which was observed in inoculated wheat samples. The modified mycotoxin, D3G was detected and quantified in both inoculated and naturally-infected samples. D3G, one of the several masked mycotoxins, is a phase II plant metabolite of the *Fusarium* mycotoxin DON [43], which can be hydrolyzed in the digestive tract of mammals, thus contributing to the total dietary DON exposure of individuals [20].

### 4.2. Culmorin (CULM), 15-Hydroxyculmorin, and 5-Hydroxyculmorin

The average value of one of the “emerging mycotoxins” culmorin (CULM) in investigated inoculated samples was 6109 µg/kg, and in naturally-infected samples, this value was 1010 µg/kg. In Norway, the concentration in naturally-infected wheat was lower at median concentrations of 100 µg/kg [44]. This concentration was much higher in durum wheat in central Italy, where it was found that *F. graminearum* produced CULM at concentrations that were very high (2500–14,000 µg/kg) [45]. It was found that naturally-contaminated Norwegian wheat, barley, and oat samples with high DON concentrations also contained CULM and hydroxyculmorins at relatively high levels [46]. In addition, CULM and various hydroxy-culmorins (5- and 15-hydroxy-culmorin) were present in the same concentration ranges as DON in naturally-contaminated grain. This was also concluded by other researchers where levels were typically positively correlated with the amount of DON [44,47]. In Croatian wheat samples in the brewing industry, DON ranged from 595 µg/kg [48] to 2723 µg/kg in wheat malt [49], while concentrations in barley were considerably lower (up to 17.6 µg/kg) [50].

In the current research along with CULM, 15-hydroxyculmorin and 5-hydroxyculmorin also occurred. According to one study [51] CULM, 15-hydroxyculmorin, 5-hydroxyculmorin, and 15-hydroxyculmoron were detected after inoculation with *F. graminearum*, showing enhanced DON toxicity to insects, impacting both growth and mortality, although according to some researchers, CULM and DON can have a synergistic effect on toxicity [52]. Recent findings indicate that CULM can suppress the activity of uridine diphosphate glucosyltransferases (UGTs) that catalyze the glycosylation of DON into the less toxic DON 3-glucoside (D3G) [15]. Compared to DON, in the current research, D3G was found in lower concentrations. All varieties had higher CULM production in inoculated samples compared to those that were naturally infected, which can result in synergistic phytotoxic effects of DON and CULM, when CULM is present at a higher concentration than DON [16], which was not the case in our research.

### 4.3. Aurofusarin–Rubrofusarin

A pioneer study identified aurofusarin, rubrofusarin, and their derivatives among the pigments [53]. It was concluded that all aurofusarin-producing organisms also have the potential to produce rubrofusarin because the latter pigment is an intermediate of the aurofusarin biosynthetic pathway [54]. This was confirmed in this research, where all samples with aurofusarin contained rubrofusarin. Similar concentrations of aurofusarin in this research were detected as in a study where researchers detected aurofusarin at 10,400–14,0000 µg/kg in Italian samples of durum wheat [54]. Mutants with the absence of the pigment aurofusarin seem to produce an increased amount of ZEN [55]. In contrast, in the current research, samples (Ficko, Pepeljuga, and Mv Karizma in the naturally-infected treatment) with the lowest production of aurofusarin showed the lowest production of ZEN.

### 4.4. Enniantins (Enns)

Mycotoxin contamination by emerging *Fusarium* mycotoxins, such as beauvericin and enniatins, represents a problem of global concern, especially in northern Europe [19,56]. The various enniatins were ranked as follows, in descending order of incidence and mean concentration: enniatin B > enniatin B1 > enniatin A1 > enniatin B2 > enniatin A > enniatin B3. Similarly, Enn analogue concentrations displayed the following gradient (EnnB1 > EnnB2 > EnnA1 > EnnB > EnnA > EnnB3) [45]. In a French study done with wheat, durum wheat, triticale, and barley, enniatin B was the toxin present in the largest amounts in all crops, followed by enniatin B1, enniatin A1, and enniatin A [57]. In other research, the various enniatins were ranked as follows, in descending order of incidence and mean concentration: enniatin B > enniatin B1 > enniatin A1 > enniatin A [19,58]. Enniatin B (trace to 4.8 μg/g) was detected in 12 samples, enniatin B1 (trace to 1.9 μg/g) was detected in eight samples, and enniatin A1 (trace to 6.9 μg/g) was detected in 10 samples [59]. In the current research, enniatin concentrations varied between varieties but were the highest in Mv Kolompos and lower in other varieties. It was expected that more enniatins were presented in naturally-infected samples due to the fact that *Fusarium* species used for inoculation do not produce enniatins.

### 4.5. Fumonisin

To date, only the fumonisins FB1 and FB2 appear to be toxicologically significant. The occurrence of FB1 in cereals, primarily maize, has been associated with serious outbreaks of leukoencephalomalacia (LEM) in horses and pulmonary oedema in pigs. However, *F. proliferatum* appears as a source of fumonisins in wheat grain, and fumonisins might, at very low levels (in the ppb range), be frequently present in wheat [60]. Fumonisins have been reported to cause diseases in humans and animals after consumption of contaminated food and feed, especially esophageal cancer [61]. In the current research, the concentrations of fumonisin B1 and B2 were in accordance with other studies where the natural occurrence of fumonisin B1 (FB1) ranged from 15–155 μg/kg, fumonisin B2 (FB2) ranged from 12–86 μg/kg, and fumonisin B3 (FB3) ranged from 13–64 μg/kg [62]. In other research, concentrations of FB1 were higher, ranging from 958 to 4906 µg/kg [63].

### 4.6. Zearalenone (ZEN), Moniliformin (MON), and Beauvericin (BEA)

*F. graminearum* is among the main species producing ZEN in Bulgarian wheat where more than half of the samples tested were contaminated with ZEN [64]. In the current research, we used a strain of *F. gramienarum,* so it was expected that higher concentrations of ZEN would occur in the inoculated samples, but as confirmed in previous research, *F. graminearum* prefers lower temperatures for the production of ZEN [65].

Moniliformin (MON) had higher concentrations in inoculated samples of Ficko than in durum wheat, which contained 45.1 µg kg^−1^ of MON in the 2013/2014 season and 171.7 µg kg^−1^ of MON in the 2012/2013 season [66].

In the current research, only one sample contained beauvericin (BEA) in trace amounts. Opposite to that in previous Italian research, BEA was detected (0.64 to 3.5 μg/g) in all investigated samples [59]. Also, BEA was detected at concentrations <10 μg/kg in field samples from Norway and Finland [19]. Although MON and BEA were not produced by the *Fusarium* species tested, they occurred in small amounts due to natural infection by other *Fusarium* species in the field.

### 4.7. Other Fusarium Metabolites

No T-2 toxin or HT-2 toxins were reported in the current investigated wheat samples compared to the notable percentages of European contaminated wheat samples [8]. Equisetin occurred sporadically in some samples due to natural infection by some other *Fusarium* species that are capable of producing it [67]. Besides *Aspergillus*, the main producers of antibiotic Y are *F. avenaceum* and *F. acuminatum*, but as *F. avenaceum* occurred in lower abundance, it was expected that antibiotic Y occurred only in one sample [68].

### 4.8. Alternaria, Aspergillus Mycotoxins and Other Metabolites

In the current research, besides *Fusarium* mycotoxins/metabolites, *Alternaria* and *Aspergillus* mycotoxins and some other metabolites were detected. This is in accordance with previous research where in field experiments, mycotoxins produced by several different *Fusarium* spp. were detected together with traces of those produced by *Alternaria* spp. [69]. Commonly, mycotoxinogenic fungi are divided into two groups: preharvest (mainly *Fusarium* species) and postharvest (mainly *Aspergillus* and *Penicillium* species) fungi.

The results for most *Alternaria* mycotoxins are in accordance with research where contamination levels in grains were <100 µg/kg and maximum concentrations were <1000 µg/kg [70]. However, the maximum observed tenuazonic acid (TeA) contamination level in wheat was 4224 µg/kg, which is a much higher concentration than that in the current research, i.e., 125 µg/kg in inoculated samples and 138 µg/kg in naturally-infected samples. The higher presence of *Alternaria* mycotoxins in naturally infected samples is expected, since the *Alternaria* spp. did not have to compete with high levels of used *Fusarium* species. Therefore, higher levels of *Alternaria* mycotoxins were documented. It is worth noting that there were statistically significantly higher levels of tentoxin and infectopyron in naturally-infected samples compared to inoculated samples (*p* = 0.03).

Kojic acid, which is produced by *Aspergillus* and *Penicillium* species [71], was observed in two inoculated samples in the current research.

## 5. Conclusions

This study detected the presence of a broad range of metabolites in wheat, and future attention should be paid not only to the mycotoxins addressed by regulations, but also to emerging and modified mycotoxins/metabolites. The results showed:

- the presence of 36 wheat mycotoxins/metabolites under field conditions in Croatia under *Fusarium* inoculation and in a natural infection environment in the harvest year 2019;
- the most abundant fungal metabolites were DON, CULM, 15-hydroxyculmorin, 5-hydroxyculmorin, and aurofusarin;
- an increase in DON in inoculated (treated) compared to naturally-infected samples;
- the absence of T2 and HT2 toxins in wheat produced commercially;
- decreased values of Enns and Fumonisins in the inoculated samples—these are produced by other *Fusarium* species that probably out-competed the *F. culmorum*/*F. graminearum* in the inoculated samples.

More wheat varieties included in the research could prove to be a better step towards identifying the mycotoxins in wheat in Croatia, which will be examined in our next investigation.

## Supplementary Materials

The following are available online at https://www.mdpi.com/2076-2607/8/4/578/s1, Figure S1: The boxplot diagrams for the statistically significant differences between two treatments (inoculated and naturally-infected) for the major *Fusarium graminearum* and *F. culmorum* metabolites. Figure S2: The boxplot diagrams for the statistically significant differences between two treatments (inoculated and naturally-infected) for the other detected metabolites.
