# Supplementary material for: Multiple Fungal Metabolites Including Mycotoxins in Naturally Infected and Fusarium-Inoculated Wheat Samples
Source: Microorganisms. 2020 Apr 17;8(4):578. doi: 10.3390/microorganisms8040578 (PMC7232504; doi:10.3390/microorganisms8040578)

Supplementary file 1.

Figure 1. The boxplot diagrams for the statistically significant differences between two treatments (inoculated and naturally-infected) for the major *Fusarium graminearum* and *F. culmorum* metabolites: deoxynivalenol (a), culmorin (b), 3-acetyldeoxynivalenol (c), 15-hydroxyculmorin (d), deoxynivalenol -3-glucoside (e) and 5-hydroxyculmorin (f). Data are presented as median, min-max and interquartile range; the $p < 0.05$ indicates statistical significance.

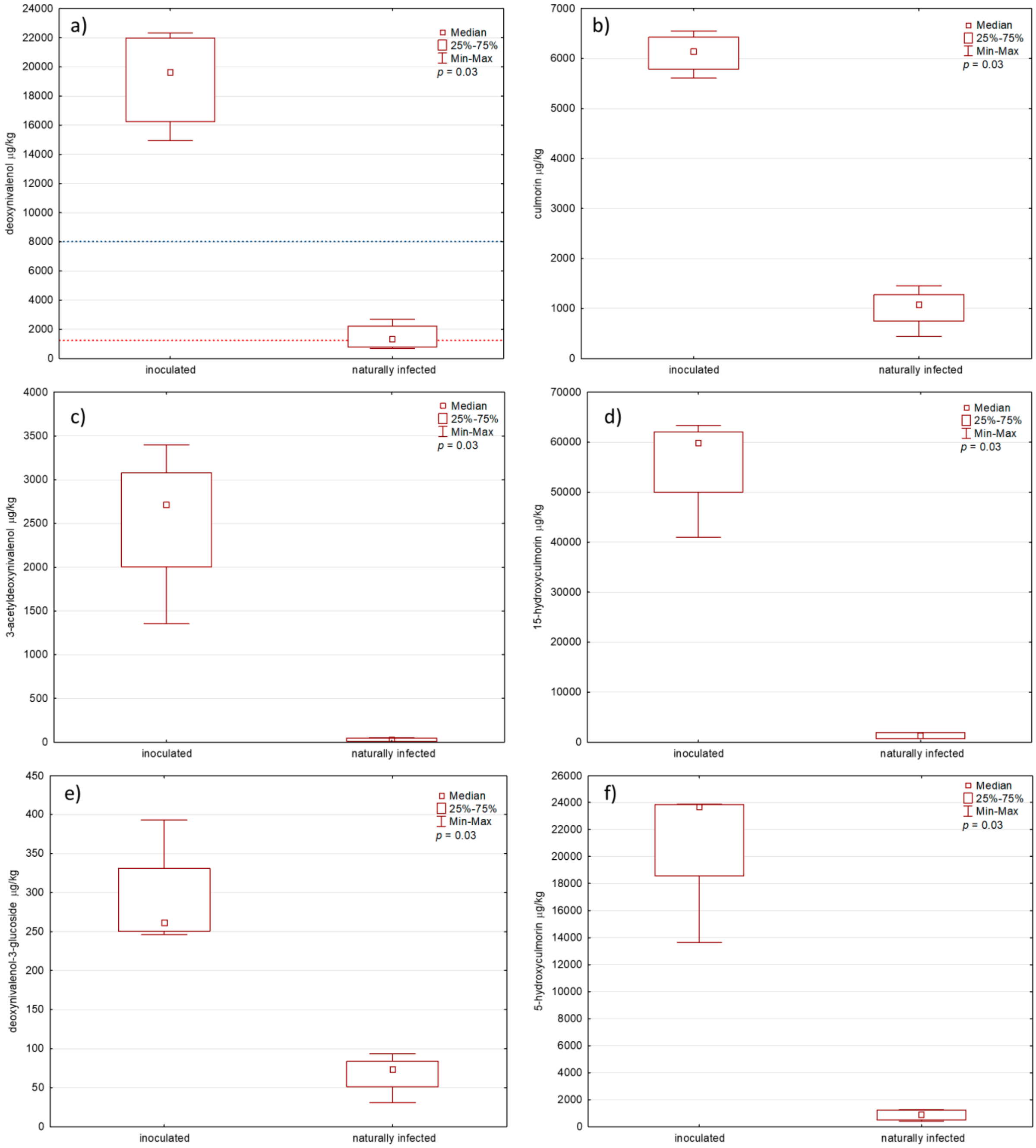

*in the Figure 1a. red dotted line represents maximum permitted level of deoxynivalenol in wheat for human consumption (1250 µg/kg) according to EC 1881/2006; while the blue dotted line represents maximum recommended concentration in wheat in animal feed (8000 µg/kg) according to EC 576/2006.

Supplementary file 2.

Figure 2. The boxplot diagrams for the statistically significant differences between two treatments (inoculated and naturally-infected) for the other detected metabolites: infectopyron (a), tentoxin (b), aurofusarin (c), rubrofusarin (d), butenolid (e), chrysogin (f) and cyclo(L-Pro-L-Val) (g). Data are presented as median, min-max and interquartil range; the $p < 0.05$ indicates statistical significance.

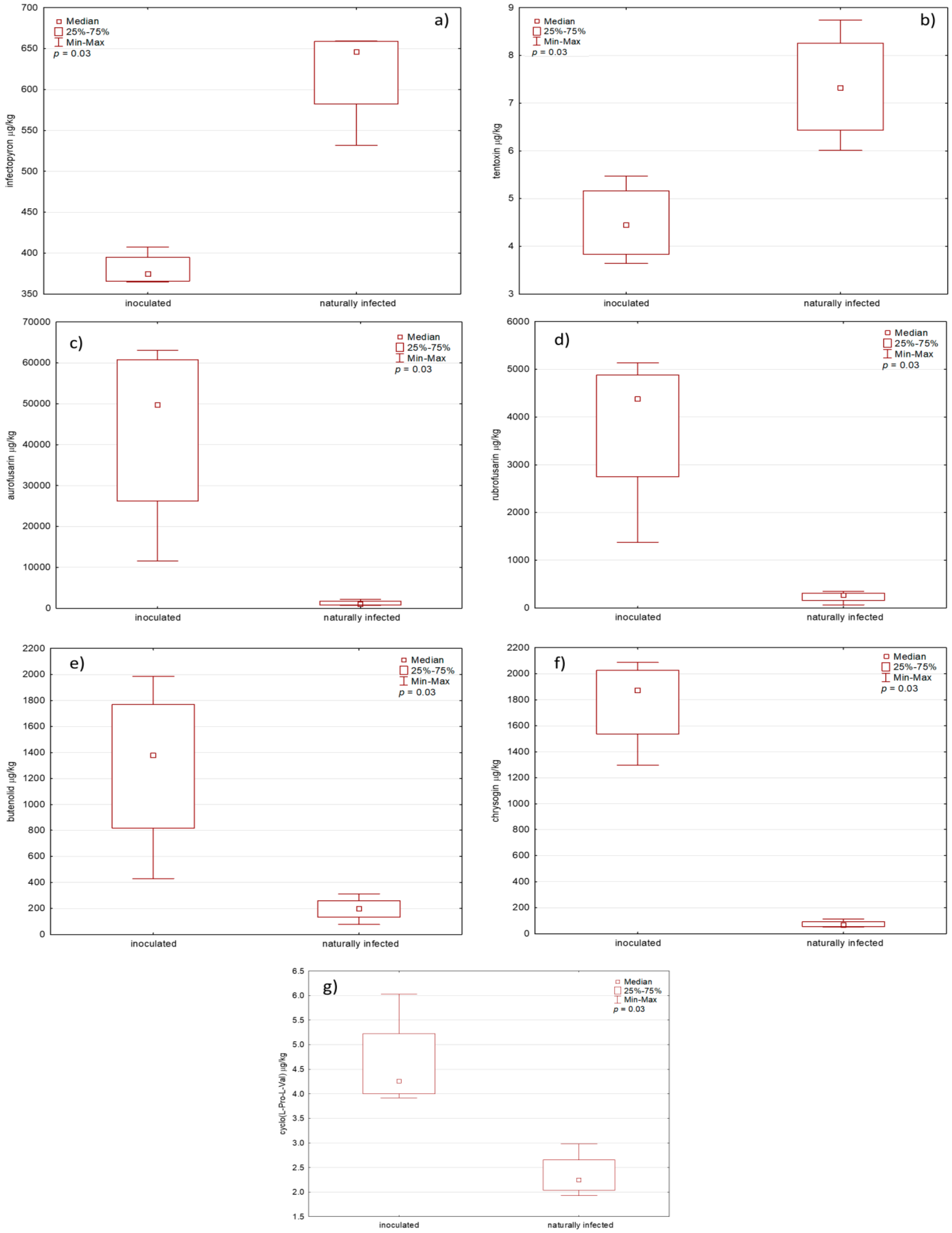

Supplement: Supplementary file 1 [file microorganisms-08-00578-s001.pdf]
